# Manipulation of Lipid Nanocapsules as an Efficient Intranasal Platform for Brain Deposition of Clozapine as an Antipsychotic Drug

**DOI:** 10.3390/pharmaceutics16111417

**Published:** 2024-11-05

**Authors:** Ahmed A. Katamesh, Hend Mohamed Abdel-Bar, Mohammed Khaled Bin Break, Shimaa M. Hassoun, Gehad Subaiea, Amr Radwan, Hadel A. Abo El-Enin

**Affiliations:** 1 Department of Pharmaceutics, College of Pharmacy, University of Ha’il, Ha’il 81442, Saudi Arabia; 2 Department of Pharmaceutics, Egyptian Drug Authority, Giza 12511, Egypt; hadelaboenin@outlook.com; 3 Department of Pharmaceutics, Faculty of Pharmacy, University of Sadat City, Sadat City 32897, Egypt; 4 Department of Pharmaceutical Chemistry, College of Pharmacy, University of Ha’il, Ha’il 81442, Saudi Arabia; 5 Medical and Diagnostic Research Centre, University of Ha’il, Ha’il 55473, Saudi Arabia; 6 Department of Pharmacology, College of Pharmacy, University of Ha’il, Ha’il 81442, Saudi Arabia; 7 Northeast Delta Branch, Department of Pharmacies, Health Insurance Organization, Mansoura 35511, Egypt; 8 Research Department, Academy of Scientific Research and Technology, Cairo 11694, Egypt; 9 Egyptian Center for Innovation and Technology Development, Cairo 11512, Egypt

**Keywords:** Intranasal, lipid nanocapsule, clozapine, antipsychotic, brain targeting

## Abstract

Background/objectives: The blood–brain barrier (BBB) significantly limits the treatment of central nervous system disorders, such as schizophrenia, by restricting drug delivery to the brain. This study explores the potential of intranasal clozapine-loaded lipid nanocapsules (IN LNCs_Clo_) as a targeted and effective delivery system to the brain. Methods: LNCs_Clo_ were prepared using the phase inversion technique and characterized in terms of size, zeta potential, entrapment efficiency (EE%), and in vitro drug release. The pharmacokinetic, safety, and pharmacodynamic effects of LNCs_Clo_ were then evaluated in a rat model through intranasal (IN) administration and compared with those of oral and intravenous (IV) Clo solutions. Results: LNCs_Clo_ were prepared using a phase inversion technique, resulting in a nanocarrier with a particle size of 28.6 ± 3.6 nm, homogenous dispersion, and high EE% (84.66 ± 5.66%). Pharmacokinetic analysis demonstrated that IN LNCs_Clo_ provided enhanced Clo brain bioavailability, rapid CNS targeting, and prolonged drug retention compared to oral and intravenous routes. Notably, the area under the curve (AUC) for brain concentration showed more than two-fold and eight-fold increases with LNCs_Clo_, compared to IV and oral solutions, respectively, indicating improved brain-targeting efficiency. Safety assessments indicated that LNCs_Clo_ administration mitigated Clo-associated metabolic side effects, such as hyperglycemia, insulin imbalance, and liver enzyme alterations. Additionally, pharmacodynamic studies showed that LNCs_Clo_ significantly improved antipsychotic efficacy and reduced schizophrenia-induced hyperactivity, while preserving motor function. Conclusions: These results highlight the potential of IN LNCs_Clo_ as a novel drug delivery system, offering improved therapeutic efficacy, reduced systemic side effects, and better patient compliance in the treatment of schizophrenia and potentially other CNS disorders.

## 1. Introduction

Psychiatric disorders, particularly schizophrenia, have become significant public health challenges, manifesting as severe behavioral and psychological disturbances that can impair daily functioning and pose substantial health risks, including chronic disability and increased suicide rates [1]. These disorders, which psychiatrists have been diagnosing for over a century, have far-reaching socioeconomic consequences, contributing to social isolation, reduced workplace productivity, and a higher incidence of chronic illnesses [2]. Their complexity is further exacerbated by comorbid conditions such as hypertension, diabetes, cardiovascular diseases, and cancer [3]. Among the treatment options for schizophrenia, clozapine (Clo) remains the most effective for managing treatment-resistant cases [4]. Clo, a second-generation antipsychotic, has demonstrated unparalleled efficacy in reducing the symptoms of treatment-resistant schizophrenia. In addition to managing recurrent suicidality and aggressive behavior, it has been employed to treat psychosis in Parkinson’s disease [5,6]. Clo’s mechanism of action is distinguished by its weak antagonism of dopamine (D2) receptors and its strong affinity for serotonin (5-HT2A/2C) receptors, which confers its superior antipsychotic efficacy [6]. However, despite its therapeutic potential, Clo’s clinical use is restricted by severe side effects, including nocturnal enuresis, sedation, gastrointestinal disturbances, metabolic complications like weight gain and diabetes), cardiovascular risks like tachycardia and orthostatic hypotension, and serious hematological conditions like agranulocytosis and neutropenia [7,8]. The risk of agranulocytosis requires regular blood monitoring, limiting patient compliance and leading to significant dropout rates during long-term treatment [9]. Furthermore, Clo has poor bioavailability (approximately 27%) when administered orally due to its extensive first-pass metabolism in the liver [10].

The delivery of antipsychotic drugs like Clo to the brain poses a major challenge due to the restrictive nature of the blood–brain barrier (BBB), which significantly limits the direct penetration of most therapeutic agents [11]. To address this challenge, advances in nanotechnology have opened new avenues for drug delivery, offering promising solutions through nanoparticle-based systems that can enhance drug bioavailability and target specific tissues, particularly the brain [12]. Various nanoparticle platforms have been explored for Clo delivery, including nanostructured lipid carriers, mixed micelles, solid lipid nanoparticles, and nanoemulsions [12,13,14,15]. Additionally, polymeric nanoparticles made from materials such as poly (lactic-co-glycolic acid) and poly-ε-caprolactone have shown enhanced nose-to-brain transport of Clo [16]. More recently, carbon dot-based nanoparticles have been investigated for their potential to improve the brain-targeting efficiency of Clo [17]. These formulations are aimed at overcoming the limitations of conventional oral or intravenous (IV) administration, which are associated with systemic side effects and poor drug targeting to the CNS [18,19].

Among the diverse range of nanotechnology-based platforms, lipid nanocapsules (LNCs) have emerged as one of the most promising and versatile drug delivery systems. LNCs consist of an oily lipid core surrounded by a polymeric shell, with surfactants embedded in their structure to enhance stability and control particle size [20,21]. The unique architecture of LNCs offers several advantages for drug delivery; they protect encapsulated drugs from premature degradation, enhance drug solubility, and facilitate controlled release over extended periods [22,23]. Furthermore, the non-toxic nature of the materials used in LNCs, coupled with their ease of large-scale production and cost-effectiveness, make them an attractive option for pharmaceutical applications [24].

LNCs have been particularly effective in overcoming the challenges associated with drug delivery to the brain [25]. Their small size and ability to cross biological barriers such as the BBB make them an ideal carrier for CNS-targeted therapeutics. The surfactant layer in LNCs, typically composed of biocompatible agents like Kolliphor^®^ HS15 and Epikuron^®^, facilitates the formation of stable nanoparticles with a uniform size distribution and prevents aggregation [26]. Moreover, LNCs have demonstrated the capacity to encapsulate lipophilic drugs like Clo, improving their solubility, enhancing brain penetration, and allowing for sustained release, thereby minimizing fluctuations in drug concentration and reducing the frequency of dosing [27,28]. The simplicity and reproducibility of the phase inversion method used to produce LNCs further bolster their suitability for clinical applications [29].

Intranasal (IN) delivery of nanoparticles, including LNCs, has garnered increasing interest as a non-invasive approach for direct drug transport to the brain [30]. By bypassing the BBB, the intranasal route facilitates the rapid onset of drug action and reduces systemic side effects [31]. This route exploits the highly vascularized and porous nature of the nasal mucosa, which allows for efficient drug absorption and direct transport to the CNS via olfactory and trigeminal nerve pathways [32].

In light of these advancements, this study seeks to optimize the use of LNCs for the IN delivery of Clo, hypothesizing that this approach will significantly enhance brain targeting while minimizing the systemic side effects associated with oral and IV routes. The specific objectives of the study are to design, develop, and evaluate an IN Clo-loaded LNCs (LNCs_Clo_) formulation, focusing on improving therapeutic efficacy, reducing adverse effects, and enhancing patient compliance.

## 2. Materials and Methods

### 2.1. Materials

Clozapine (Clo), Kolliphor^®^ HS15, acetonitrile (HPLC grade), ethanol absolute (HPLC grade), Triton X-100, and ketamine were purchased from Sigma Aldrich (St. Louis, MO, USA). Labrafac was kindly gifted from Gattefossé (Berkshire, UK). Epikuron 200^®^ was purchased from Cargill (Minneapolis, MN, USA).

### 2.2. Method

#### 2.2.1. Clozapine-Loaded Lipid Nanocapsule Formulation

Lipid nanocapsules (LNCs_Clo_) loaded with clozapine (Clo) were prepared using a phase inversion method. Based on preliminary studies, an oil surfactant mixture containing Labrafac and Kolliphor^®^ HS15 at a 1:4 *w*/*w* ratio was combined with Epikuron 200^®^ (1.5% *w*/*w*). The mixture was agitated using a magnetic stirrer (CB302 ceramic plate, Jenway Ltd., London, UK) at 500 rpm for 30 min. Clo was then dissolved in the oil surfactant mixture at a concentration of 10% *w*/*w*. To the resulting dispersion, an equal volume of sodium chloride solution (1.75%) was added dropwise. The obtained LNCs_Clo_ formulation was subjected to three thermal-cooling cycles, each alternating between 70 °C and 40 °C, while maintaining continuous magnetic stirring at 500 rpm. During the final cycle, the mixture was diluted with 10 mL of cold deionized water (4 °C) to facilitate the fabrication of the LNCs_Clo_ [21]. The obtained LNCs_Clo_ was purified using Amicon^®^ Ultra centrifugal filter units (15 mL, 30 kDa Merck Millipore, Darmstadt, Germany) at 14,000 rpm for 45 min at 4 °C to remove unentrapped Clo. The collected LNCs_Clo_ was kept at 4 °C for further analysis.

#### 2.2.2. Determination of Particle Size and Zeta Potential

The particle size and polydispersity index (PDI) of the prepared LNCs_Clo_ were measured by using dynamic light scattering (DLS) and with a Nanosizer ZS Series (Malvern Instruments, Southborough, MA, USA). The surface charge expressed as zeta potential was determined using electrophoresis. The LNCs_Clo_ was diluted with deionized water (1:100 *v*/*v*) and all measurements were calculated as an average of 20 runs, each run was completed in triplicate at 25 °C [33].

#### 2.2.3. Determination of Entrapment Efficiency Percentage and Loading Efficiency

The Clo entrapment efficiency percentage (EE%) was determined directly by dissolving the LNCs_Clo_ using 10% Triton X-100 (0.5 mL). The amount of Clo released was then quantified using a previously established HPLC method. Briefly, the HPLC system was composed of a Dionex^TM^ system (Thermo Scientific^TM^, Waltham, MA, USA) equipped with a C18 column (250 × 4.6 mm, 5 μm, Hypersil^®^ ODS, Thermo Scientific^TM^, Waltham, MA, USA). The chromatographic separation was conducted using a mobile phase composed of potassium dihydrogen orthophosphate (10 mM, pH 3.0) and acetonitrile at a ratio of 65:35 (*v*/*v*) and a flow rate of 1 mL/min. The UV detector was set at 292 nm [12]. The coefficient of determination (R^2^) of the Clo calibration curve in PBS (pH 7.4) containing Triton X-100 (10%) in the concentration range of 10−500 ng/mL was 0.9992 (Figure S1), and the respective limit of detection (LOD) and quantification (LOQ) were 7 and 10 ng/mL. The coefficients of variation (CV)% ranged from 2.4 to 4.7%, and the accuracy for Clo determination was within the range of 1.3−4.3% with a mean% drug recovery of 98.1 ± 2.05%. The retention time of Clo separation was 3.8 min.

The EE% was calculated according to the following equation:

(1) $$\mathrm{EE}\%=\frac{amount\;of\;Clo\;inside\;the\;pelletes}{Total\;amount\;of\;Clo\;added}\;\times \;100$$

The loading efficiency (LE%) was calculated as follows:

(2) $$\mathrm{LE}\%=\frac{Weight\;of\;Clo\;entrapped\;into\;LNCs}{Total\;weight\;of\;{LNCs}_{Clo}}\;\times \;100$$

#### 2.2.4. Transmission Electron Microscopy

Morphological structures of the LNCs_Clo_ were investigated using a Transmission Electron Microscope (TEM) (JEOL JEM1230, Tokyo, Japan) as described elsewhere [29]. Briefly, the LNCs_Clo_ was placed onto a carbon-coated copper 300-mesh grid and left to stand for 10 min. Excess liquid was then removed using filter paper. The sample was stained with a drop of 1% phosphotungstic acid, allowed to dry for 5 min, and subsequently imaged using TEM at an accelerating voltage of 200 kV.

#### 2.2.5. In Vitro Drug Release

The in vitro drug release from the LNCs_Clo_ formulation was evaluated using the dialysis membrane technique. In brief, a measured volume of the optimized LNCs_Clo_ formulation, equivalent to 2 mg of Clo, was diluted with 1 mL of simulated nasal fluid (SNF, pH 7.4). The SNF was prepared by dissolving sodium chloride (2.1925 g), calcium chloride (0.145 g), and potassium chloride (0.745 g) into 250 mL of double-distilled water [34]. The mixture was subsequently placed into a dialysis membrane with a molecular weight cutoff of 10,000 Da. The sealed dialysis bag was immersed in 25 mL of SNF (pH 7.4) and maintained at 35 ± 0.5 °C, the reported nasal mucosa temperature [35]. The release was conducted under continuous stirring at 100 ± 0.1 S/min using thermostatic Shaker (Daihan Labtech Shaker Water Bath, LSB 030S, Seoul, South Korea). At predetermined time intervals, 0.2 mL aliquots were withdrawn from the solution and replaced with fresh SNF. The drug content in the collected samples was quantified using a previously validated HPLC method.

#### 2.2.6. Shelf-Life Stability

The obtained LNCs_Clo_ dispersion was stored at 4 °C for 7 days. The particle size, zeta potential, and EE% were evaluated after 1, 3, 5, and 7 days as previously described.

#### 2.2.7. Pharmacokinetic Profiling, Safety Assessment, and Therapeutic Efficacy

##### Animals

Adult male Sprague-Dawley rats (three months old and weighing 200 g ± 10%) were housed under regular conditions of food, filtered water, 12 h light/dark cycle, constant temperature, and humidity. All animal studies were approved by the ethical committee of the Faculty of Pharmacy, University of Sadat City (approval number; RERC-FOP-USC-24-02-06).

##### Pharmacokinetic Studies

One hundred forty-four rats were randomly divided into three groups: the first and second groups received either the Clo solution via intravenous (IV) injection or oral administration, respectively, while the last group received the LNCs_Clo_ intranasally (IN). The Clo solution (100 µL), either IV or oral, was prepared by dissolving Clo in 2.5 μL of 1.2 N HCl, then diluted with normal saline [36]. The composition of the IN LNCs_Clo_ administered was Labrafac (5.4 mg/kg), Kolliphor HS15 (21.615 mg/kg), and Epikuron 200 (0.45 mg/kg). A dose of 2.5 mg/kg was administered either through injection into the tail vein, via an oral route, or by instilling 10 µL of the LNCs_Clo_ into each nostril. An oral gavage method was employed to ensure that the rats completely ingested the oral dose of the drug. Using a flexible gavage needle, the precise dose was carefully delivered directly into the rat’s stomach, minimizing the risk of spillage or partial ingestion [36]. The rats were monitored briefly after administration to confirm complete ingestion and check for any potential regurgitation. Blood samples were collected via cardiac on heparinized tubes at specified time intervals. Following this, the rats were euthanized, and their brains were carefully removed. The excised brains were immediately placed in ice-cold phosphate-buffered saline (PBS, pH 7.4) and homogenized at 25% *w/v* in PBS. The blood samples and the homogenates were centrifuged at 9000 rpm for 15 min. Plasma and brain homogenate were then mixed with an equal volume of acetonitrile to precipitate proteins. Following vortex mixing and centrifugation at 12,000 rpm for 10 min, the samples were stored at −80 °C for further analysis [37]. Clo concentrations in both plasma and the brain were quantified using a previously established HPLC method [12].

Pharmacokinetic parameters, including maximum drug concentration (C_max_), time to reach C_max_ (T_max_), area under the concentration-time curve (AUC_0–480_ and AUC_0–∞_), mean residence time (MRT), elimination rate constant (K_el_), and absolute as well as relative bioavailability, were calculated from the collected plasma and brain samples using PK Solver software, for Microsoft 2010. Additionally, drug-targeting efficiency (DTE%) and direct-transport percentage (DTP%) were determined using the following equations [37]:

DTE = (AUC brain/AUC plasma) IN/(AUC brain/AUC plasma) IV × 100(3)

DTP% = BIN − BX/BIN × 100(4)

where BX = BIV/PIV × PIN;

- BX is the brain AUC 0–480 fraction contributed by systemic circulation, following IN administration;
- BIV is the brain AUC_0–480_ following IV administration; PIV is the plasma AUC_0–480_ following IV administration;
- BIN is the brain AUC_0–480_ following IN administration;
- PIN is the plasma AUC_0–480_ following IN administration.

##### Safety Assessment Study

Thirty rats were randomly assigned into three equal groups for an 8-week study. The control group received oral administration of PBS (pH 7.4) once daily. The second group was treated with LNCs_Clo_, administered intranasally at a dose of 2.5 mg/kg per day. The third group received an oral Clo solution at the same dosage and frequency as the LNCs_Clo_ group. Throughout the study, the animals were fasted for 12 h each week before blood collection from the tail veins. Each 2 mL blood sample was split into two portions: one was treated with heparin and centrifuged at 9000 rpm for 15 min at 4 °C to separate plasma, while the other was allowed to clot at room temperature before centrifugation to obtain serum. Both plasma and serum were subsequently stored at −80 °C for later analysis. Plasma glucose levels were measured using a colorimetric glucose assay kit (Cell Biolabs, Inc., San Diego, CA, USA), and plasma insulin concentrations were determined via an ELISA using a rat insulin enzyme immunoassay kit (Invitrogen, Paisley, UK) and an ELISA microplate reader (Chro-Mate-4300, Awareness Technology, Inc., Jensen Beach, FL, USA). Additionally, serum triglyceride levels, as well as alanine aminotransferase (ALT) and aspartate aminotransferase (AST) concentrations, were assessed using the appropriate colorimetric kits (Sigma-Aldrich, Dorset, UK), following the manufacturer’s protocols.

##### Paw Placement Test

A Perspex platform measuring 30 × 30 cm with a height of 20 cm was used to conduct the paw retraction test. The platform featured four holes: two smaller ones (4 cm in diameter) for the forelimbs and one larger hole (5 cm in diameter) for the hindlimbs and tail. Rats were divided into four groups, each consisting of six rats. The negative control group received oral PBS (pH 7.4). After 30 min, the rats’ forelimbs were gently placed into the corresponding holes, followed by their hindlimbs. The forelimb retraction time (FRT) and hindlimb retraction time (HRT) were recorded, with FRT representing the time taken by the rat to withdraw a single forelimb and HRT representing the time to withdraw a single hindlimb. The minimum recorded time was 1 s, and the maximum time was 30 s. The paw test was conducted three times: once following IV Clo solution, once after oral administration, and finally after IN administration of the LNCs_Clo_ dispersion [38].

##### Pharmacodynamic Study

Fifty rats were evenly distributed across ten cages for this experiment. Group 1, serving as the healthy control group, received daily intraperitoneal (i.p.) injections of 0.2 mL PBS (pH 7.4) for one week. Schizophrenia was induced in the remaining forty rats via an i.p. injection of ketamine at a dose of 25 mg/kg. Group 2 (*n* = 10) was kept as an untreated positive control. Groups 3, 4, and 5 were administered a daily dose of 2.5 mg/kg of either the IV Clo solution, oral Clo solution, or IN LNCs_Clo_, respectively, for seven consecutive days. To assess locomotor activity, the open-field test was performed. Each rat was placed in the center of an open-field apparatus (AccuScan Instruments, Columbus, OH, USA) measuring 40 × 40 × 30 cm, divided into 16 squares of 10 × 10 cm each. After a 2-h acclimatization period in their home cages in the testing room, individual rats were gently placed at one of the apparatus corners facing the center. The number of squares crossed by each rat during ambulation was recorded to quantify locomotor activity, and the total ambulatory distance, reflecting overall motor activity, was monitored over a 60-min period [38].

### 2.3. Statistical Analysis

All in vitro experiments were repeated thrice, and the data were reported as mean ± SD. In vivo pharmacokinetic study results were reported as the mean ± standard error (SE) of six replicates. The pharmacokinetic parameters were estimated with the PK-solver software version 2. The results of safety and pharmacodynamic studies were expressed as a mean of ten animals ± SE. The ANOVA test, followed by the Tukey HSD test, was used to compare the different parameters between groups, where *p* < 0.05 was considered significant.

## 3. Results and Discussion

### 3.1. Physicochemical Characterization of LNCs-Loaded Clo Formulation

The LNCs_Clo_ formulation is composed of the core and coat; the lipophilic core is primarily composed of Labrafac, an oil that encapsulates the Clo, providing a hydrophobic environment for drug loading. The coating shell consists of Kolliphor HS15 and Epikuron 200, which act as surfactants, stabilizing the LNCs structure by reducing surface tension and forming a protective layer around the lipid core [39]. In this study, the LNCs_Clo_ was prepared using the phase inversion technique, a simple and efficient method that reduces both energy consumption and reliance on organic solvents [40]. The phase inversion temperature (PIT) is the temperature at which the emulsion undergoes a phase transition from a water-in-oil to an oil-in-water emulsion. This temperature is crucial for forming stable LNCs, as it allows the controlled solidification of the lipid core and encapsulation of the drug within the nanocapsule structure [39]. The process comprises two primary steps. Initially, oil and surfactants are mixed with sodium chloride to form a primary emulsion. Subsequently, the mixture is heated above the phase inversion temperature (70 °C), resulting in a water-in-oil emulsion. Upon cooling the mixture below the phase inversion temperature (40 °C), it transitions to an oil-in-water emulsion. The final step involves introducing cold water, which disrupts the emulsion system irreversibly, leading to the solidification of the lipid into a stable nanocapsule shell [21]. This process not only enhances the rigidity of the shell and prevents the coalescence of droplets but also contributes to the stabilization of LNCs suspensions at room temperature [41]. Additionally, by controlling salinity levels, the encapsulation process is refined, allowing for the inclusion of more drug content with minimal degradation over a short heating period [42,43].

Table 1 presents the in vitro physicochemical characteristics of the produced LNCs_Clo_. The particle size was determined to be 28.6 ± 3.6 nm, likely a result of the presence of Kolliphor^®^ HS15 and Epikuron 200^®^, which effectively reduce the oil’s interfacial tension and contribute to the reduction of globule size [21]. A PDI of 0.18 ± 0.02 indicates a homogeneous, monodisperse nanoparticle system. The LNCs_Clo_ formulation also exhibited a negative zeta potential of −15.9 ± 1.9 mV. Furthermore, the Clo EE% was measured at 84.66 ± 5.66%, which can be attributed to the surfactant components enhancing the solubility and incorporation of the drug within the lipid core [41]. These properties underscore the efficiency of the LNCs_Clo_ in improving the solubility and stability of the encapsulated drug. The LNCs_Clo_ formulation promotes Clo stability through several mechanisms inherent to its LNCs structure. The encapsulation of Clo within a lipid core, protected by a surfactant shell, effectively shields the drug from external environmental factors, such as oxidation, which is a common cause of Clo instability [44]. The lipid core, primarily composed of Labrafac, provides a hydrophobic environment that stabilizes Clo by minimizing the risk of oxidation. Additionally, the surfactants Kolliphor HS15 and Epikuron 200 in the LNCs_Clo_ formulation create a stable outer shell around the lipid core. This shell not only prevents aggregation and protects against physical degradation but also reduces the potential for Clo to interact with destabilizing agents in the biological environment. The loading efficiency of Clo in the LNCs, recorded at 17.65 ± 2.11%, reflects an effective encapsulation rate for this formulation. These results are in agreement with previous studies on loading different drugs in LNCs [45]. This moderately high value suggests that the formulation process and lipid selection were favorable for incorporating Clo into the LNCs. Achieving a high LE% is crucial for ensuring adequate therapeutic dosing, reducing the administration frequency, and enhancing patient compliance, especially given Clo’s therapeutic importance [46].

**Figure 1:**
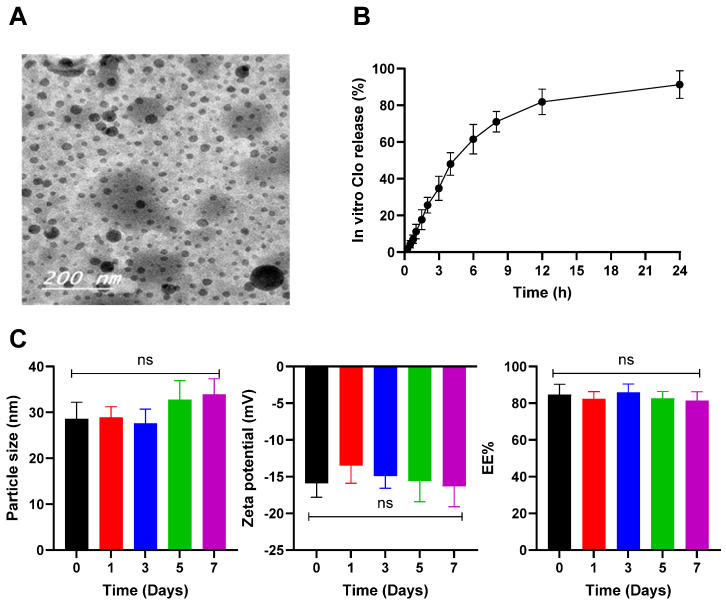
In vitro characterization of the LNCs_Clo_. Morphological characterization of the prepared LNCs_Clo_ using transmission electron micrography (**A**). The LNCs_Clo_ appeared as a spherical nonaggregate nanostructure. In vitro release profile of Clo from the LNCs_Clo_ in simulated nasal fluids at 35 °C (**B**). Drug release from the LNCs is measured by dialyzing the LNCs_Clo_ against simulated nasal fluids (pH 7.4). Drug concentration in the dialysate is assessed by HPLC. Datapoints represent mean and SD (*n* = 3). (**C**) Effects of storage at 4 °C on the LNCs_Clo_ particle size, zeta potential, and EE%. One-way analysis of variance (ANOVA) was used to compare the different parameters between groups, followed by the Tukey HSD test; ns is nonsignificant.

Figure 1A shows the transmission electron microscopy (TEM) image of the obtained LNCs_Clo_, revealing spherical particles with a relatively uniform size distribution. This is consistent with the particle size of the LNCs size measured using DLS and aligns with low PDI values commonly reported in such formulations. The in vitro release of Clo from the LNCs was monitored over a 24-h period, and the results are presented in Figure 1B. The release profile of Clo from the lipid LNCs exhibited a biphasic pattern, characterized by an initial burst release followed by a sustained and controlled release phase. In the initial burst phase, approximately 48 ± 6.17% of the drug was released within 4 h. The burst release can be attributed to the dissolution of Clo adsorbed on or near the surface of the LNCs, a common characteristic of lipid-based systems. A sustained release phase followed, reaching 81.87 ± 6.93% by 12 h and 91.33 ± 7.54% by the end of 24 h. This sustained release pattern suggests that the LNCs efficiently encapsulates Clo within the lipid matrix, allowing for controlled and prolonged drug release. The extended-release profile could potentially maintain therapeutic drug levels over time. Moreover, the limited release of the LNCs_Clo_ in the nasal cavity may, in fact, serve as an advantage by promoting the uptake of the intact LNCs rather than the free drug via endocytosis [47]. This particulate uptake mechanism facilitates direct translocation of the LNCs across the nasal epithelium, potentially enhancing drug delivery to the brain via pathways such as olfactory or trigeminal nerve routes. Consequently, the LNCs can bypass systemic circulation initially, allowing for localized brain delivery with minimal systemic exposure.

The LNCs_Clo_ preserved its in vitro characters, and no significant change in the particle size, zeta potential, or EE% was seen after 7 days of storage at 4 °C at different time intervals (Figure 1C) (*p* > 0.05). This good colloidal stability may be attributed to the zeta potential value that gave electrostatic stabilization. Additionally, the presence of surfactants provides steric stabilization of the LNCs_Clo_ and avoids aggregation [48].

### 3.2. Pharmacokinetics Profile of Clo Following IN Administration of the Prepared LNCs_Clo_

**Figure 2:**
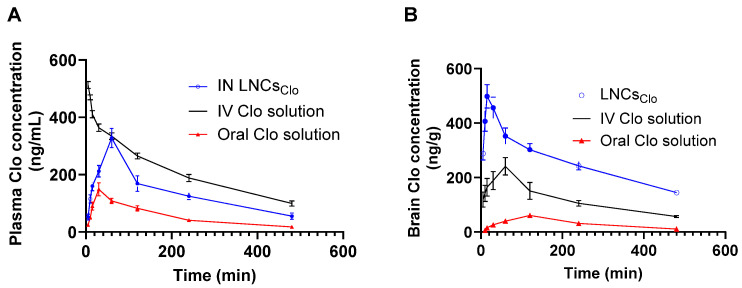
Clozapine concentrations in rat (**A**) plasma and (**B**) brain tissues, after administration of various formulations. Animals received a dose of 2.5 mg/kg of Clo either via IN LNCs_Clo,_ IV solution, or oral solution. At each time point, 6 animals were sacrificed from each group, and the concentration of Clo in plasma and brain tissues was quantified using HPLC. A significantly higher brain Clo concentration was observed at all time points following IN administration of LNCs_Clo_ compared to IV and oral solutions (*p* < 0.05). Datapoints represent mean ± SE (*n* = 6). The ANOVA test, followed by the Tukey HSD test, was used to compare the different parameters between groups, where *p* < 0.05 was considered significant.

The pharmacokinetic profiles of Clo following IN administration via LNCs_Clo_, IV solution, and oral solution were analyzed in both plasma and brain tissues (Figure 2A,B). The results indicate significant differences in drug absorption, distribution, and elimination across the different routes of administration. IN LNCs_Clo_ showed a significantly higher C_max_ than the oral Clo solution with respective values of 327.78 ± 33.66 ng/mL and 148.52 ± 22.54 ng/mL (*p* < 0.05). As expected, the IV solution showed the highest AUC_0–480min_ (1677.23 ± 89.98 ng·h/mL) compared to 1101.8 ± 121.31 ng·h/mL and 437.93 ± 46.00 ng·h/mL for IN LNCs_Clo_ and oral Clo solution, respectively (Table 2). The respective T_max_ values of IN LNCs_Clo_ and oral Clo solution were 60 min and 30 min, respectively. This could be attributed to the controlled release of Clo from the prepared LNCs (Table 2). An almost 2.5-fold increase in the bioavailability of LNCs_Clo_ was observed over the oral solution. This could be attributed to the extensive first-pass metabolism of Clo following oral administration. The calculated Kel values for IN LNCs_Clo_ and IV Clo solution were 0.18 ± 0.02 h^−1^ and 0.15 ± 0.01 h^−1^, respectively, indicating relatively similar rates of drug elimination from the plasma. On the other hand, the Kel of Clo following oral administration was 0.25 ± 0.01 h^−1^, which was significantly higher than that following either the IN or IV route (*p* < 0.05) (Table 2). A higher Kel indicates that Clo is eliminated from the body more rapidly when administered orally compared to the IN and IV routes. This accelerated elimination could be attributed to the extensive first-pass metabolism associated with oral administration, where a portion of clozapine is metabolized in the liver before reaching systemic circulation. In brain tissue, IN administration also resulted in the highest C_max_ (498.09 ± 43.03 ng/g), which was more than double that of the IV solution (241.39 ± 32.34 ng/g) and eight times higher than the oral solution (60.62 ± 6.78 ng/mL). Similarly, AUC_0–480min_ was the highest for LNCs_Clo_ (2036.45 ± 120.03 ng·h/mL), followed by the IV solution (951.78 ± 115.41 ng·h/mL) and the oral solution (248.33 ± 42.21 ng·h/mL). The T_max_ for LNCs_Clo_ was 15 min, suggesting rapid delivery to the brain, compared to 60 min for the IV solution and 120 min for the oral solution. The results from this study highlight the clear advantage of LNCs_Clo_ over both IV and oral routes. In pharmacokinetic studies assessing IN drug delivery, Drug DTE% and DTP% are essential for quantifying the extent of direct nose-to-brain drug transport. DTE% measures the relative efficiency of drug delivery to the brain via IN administration compared to IV administration. A high DTE% indicates that a substantial proportion of the drug is effectively targeted to the brain, bypassing the BBB more efficiently than with systemic administration alone. In contrast, DTP% specifically quantifies the fraction of the drug reaching the brain directly from the nasal cavity, independent of the systemic circulation and without relying on BBB penetration. Thus, a high DTP% indicates that a considerable amount of the drug is delivered directly to the brain through nasal pathways rather than systemic absorption [49]. The high DTE% (325.71%) and DTP% of 69.29%, indicate significant transport via the IN route. K_el_ was the lowest for LNCs_Clo_, indicating prolonged drug retention in brain tissue compared to the IV and oral solutions (Table 2). These results underscore the potential of LNCs as effective carriers for IN Clo delivery, offering enhanced brain targeting and bioavailability. These findings align with previous studies that have reported the benefits of IN drug delivery for CNS disorders. Clo had been successfully loaded in different nanocarriers. A nanoemulsion-based in situ gel for Clo demonstrated enhanced bioavailability through IN administration [15]. Similarly, **Lombardo and coworkers** compared freeze-dried PCL and PLGA nanoparticles, both showing promise in facilitating nose-to-brain delivery [16]. Additionally, the use of carbon dot-based nanoparticles revealed increased Clo availability in the brain via IN pathways [17]. In addition, the work by da Costa Güllich et al. (2015) further supports the benefits of nanoencapsulation, showing that Clo linked to nanocapsules significantly minimizes tissue damage and oxidative stress in the brain [50]. This study found that nano-encapsulated Clo reduced lipid peroxidation and protein carbonylation—markers of oxidative damage—compared to free Clo. Moreover, nanoencapsulation helped decrease DNA damage in brain tissue, suggesting a neuroprotective effect likely due to the controlled and targeted release provided by nanocapsules [50]. Herein, nasal administration of LNCs_Clo_ was observed to offer a direct route to the brain, bypassing the first-pass metabolism and, thus, enhancing Clo’s bioavailability in brain tissues. This route may reduce systemic exposure, minimizing the oxidative stress typically associated with Clo. By leveraging the lipid-based encapsulation, LNCs_Clo_ facilitates a sustained and controlled release of clozapine, potentially lowering the formation of reactive metabolites responsible for tissue and biomolecular damage. Together, these results indicate that nasal LNCs_Clo_ delivery enhances brain targeting and mitigates oxidative and tissue damage, aligning with the protective effects.

### 3.3. Safety Profile of Clo Following IN Administration of the Prepared LNCs_Clo_

**Figure 3:**
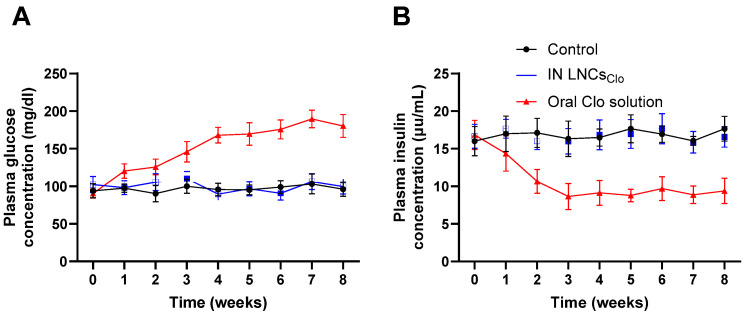
Effect of clozapine on plasma glucose level (**A**) and insulin levels (**B**). Animals received a dose of 2.5 mg/kg/day of Clo either via IN LNCs_Clo_ or an oral solution for 8 weeks. Every week, the fasting plasma glucose and insulin levels were measured. A significantly higher plasma glucose concentration and lower plasma insulin were observed at all time points following oral administration of Clo compared to the IN LNCs_Clo_ (*p* < 0.05). Datapoints represent mean ± SE (*n* = 10). The ANOVA test, followed by the Tukey HSD test, was used to compare the different parameters between groups, where *p* < 0.05 was considered significant.

The results of the safety study comparing the impact of IN LNCs_Clo_ and the oral Clo solution on plasma glucose levels demonstrated distinct patterns in glucose regulation over time (Figure 3A). The control group maintained relatively stable plasma glucose levels throughout the study, with minor fluctuations from 90.24 ± 10.98 mg/dL to 100.11 ± 9.34 mg/dL. In the intranasal LNCs_Clo_ group, plasma glucose levels began at 102.36 ± 10.69 mg/dL and showed slight variations, reaching 105.69 ± 11.36 mg/dL at week 2 and 110.36 ± 9.65 mg/dL at week 3, indicating a stable response with no significant increases till the end of the study. In contrast, the oral Clo solution group exhibited a marked rise in plasma glucose levels, starting at 90.5 ± 5.98 mg/dL and increasing significantly to 145.98 ± 13.65 mg/dL by week 3, peaking at 198.69 ± 11.69 mg/dL by week 8 (*p* < 0.05). The pronounced increase in plasma glucose observed in the oral Clo solution group aligns with previous reports linking the oral Clo solution to hyperglycemia and other metabolic disturbances [51,52,53]. These findings suggest that IN LNCs_Clo_ administration may offer a more favorable metabolic profile, with less impact on glucose regulation compared to the oral formulation. Figure 3B shows the effects of IN LNCs_Clo_ and the oral Clo solution on plasma insulin levels in rats over an 8-week period. The control group exhibited stable insulin levels throughout the study, with mean insulin concentrations ranging between 16.02 ± 1.95 μU/mL and 17.66 ± 1.65 μU/mL from week 0 to week 8. Similarly, rats treated with IN LNCs_Clo_ exhibited an almost similar plasma insulin level over the study period from 16.65 ± 1.58 μU/mL to 16.58 ± 1.36 μU/mL. In contrast, the groups treated with oral Clo solution exhibited a progressive reduction in plasma insulin concentrations, with insulin levels declining from 16.85 ± 1.95 μU/mL at baseline to 9.39 ± 1.69 μU/mL by week 8. The results of this study reveal significant differences in the impact of IN LNCs_Clo_ and the oral clozapine solution on plasma insulin levels in rats over an 8-week treatment period (*p* < 0.05). The reduction in the plasma insulin level in the oral Clo solution group was notably steeper compared to the IN group, indicating a potentially stronger systemic effect on insulin regulation through oral administration. The reduction in insulin levels observed in the oral Clo solution groups aligns with existing literature on the metabolic effects of Clo, a second-generation antipsychotic known to disrupt glucose metabolism [51,54,55]. Clo has been associated with the development of insulin resistance and an increased risk of type 2 diabetes, particularly with chronic use [56,57]. The observed decrease in insulin levels may be attributed to Clo’s influence on central insulin regulation, possibly affecting the hypothalamic pathways that control glucose homeostasis [58,59]. The more pronounced reduction in the oral Clo solution group compared to the IN group is of particular interest. In this study, the insignificant effect of IN LNCs_Clo_ on the plasma insulin level observed could suggest that IN administration might mitigate some of Clo’s adverse metabolic effects.

**Figure 4:**
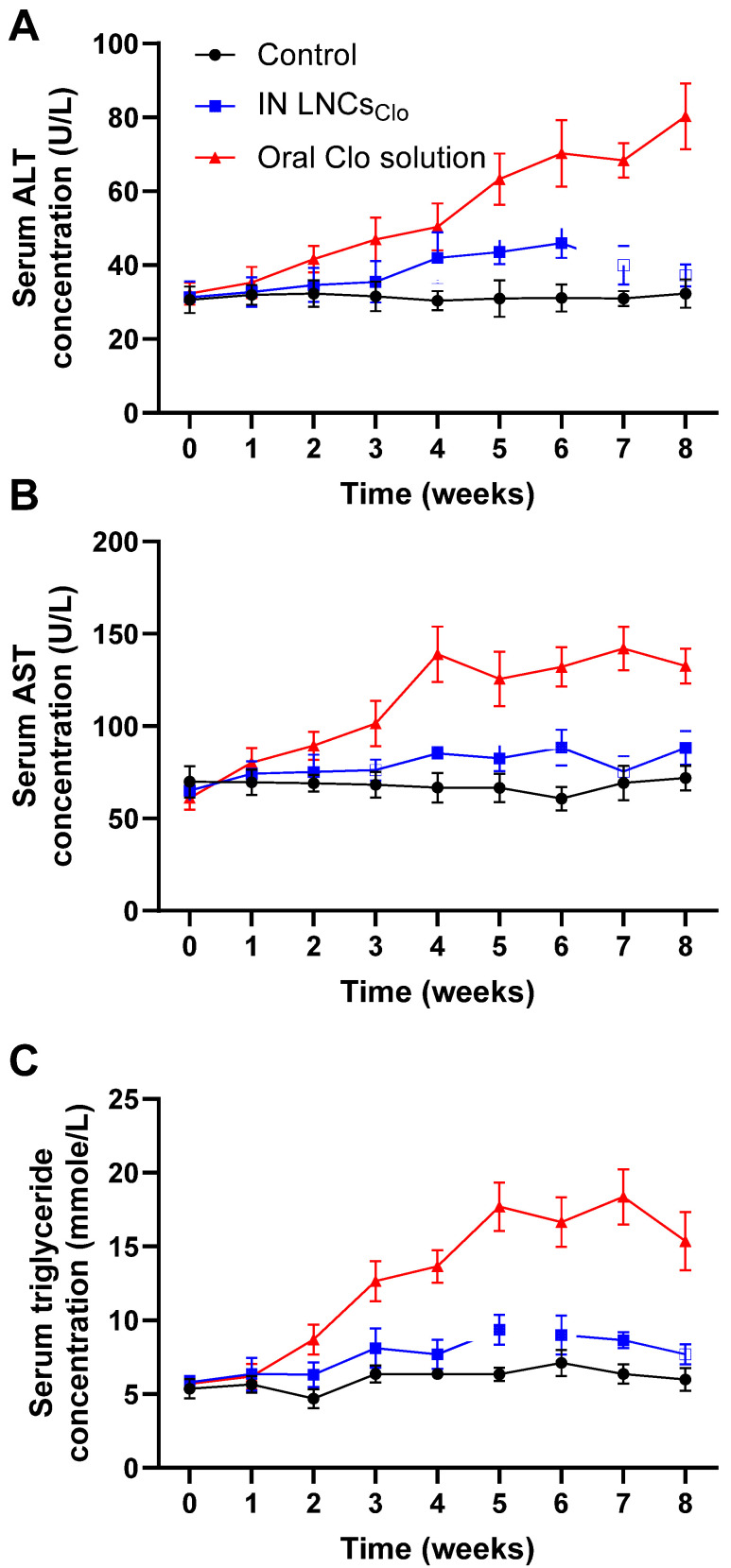
Effect of clozapine on serum (**A**) ALT, (**B**) AST and (**C**) triglyceride levels. Animals received a dose of 2.5 mg/kg/day of Clo either via IN LNCs_Clo_ or an oral solution for 8 weeks. Every week, fasting serum ALT, AST, and triglyceride levels were measured. Significantly higher serum ALT, AST, and triglyceride levels were observed at all time points following oral administration of Clo compared to IN LNCs_Clo_ (*p* < 0.05). Datapoints represent mean ± SE (*n* = 10). The ANOVA test, followed by the Tukey HSD test, was used to compare the different parameters between groups, where *p*< 0.05 was considered significant.

Figure 4A reveals the effects of IN LNCs_Clo_ and the oral Clo solution on serum ALT levels in rats over an 8-week period. Serum ALT levels were measured weekly, with the control group serving as the baseline for normal liver enzyme function. Throughout the study, the control group maintained consistent ALT levels, ranging between 30.65 ± 3.69 U/L to 32.33 ± 3.87 U/L. In the IN LNCs_Clo_ group, ALT levels remained within a relatively narrow range, peaking in the sixth week with a mean ALT level of 45.93 ± 4.02 U/L. Over the remaining two weeks, ALT levels stabilized near baseline, with week 8 showing an ALT concentration of 37.24 ± 3.04 U/L, which is comparable to the control group. The oral Clo solution group, however, demonstrated a more significant increase in ALT levels over time (*p* < 0.05). By the third week, ALT levels had risen to 46.98 ± 5.95 U/L, continuing to significantly increase to 80.33 ± 8.98 U/L by the last week (*p* < 0.05). This substantial elevation in ALT levels, a key marker of liver health, suggests a more pronounced impact of oral Clo solution on liver enzyme activity, indicating potential liver stress. The significant rise in ALT levels observed in the oral Clo solution group is consistent with previous reports of Clo-associated hepatotoxicity [60].

The increase in ALT levels over the 8-week period suggests that oral Clo solution exerts a considerable impact on liver function, possibly due to systemic drug exposure and metabolism. The effects of IN LNCs_Clo_ and the oral Clo solution on the AST level in rats over a period of 8-week period are displayed in Figure 4B. Serum AST levels were measured weekly, with the control group serving as the baseline for normal liver enzyme function. Over the course of the experiment, the control group exhibited stable AST levels, fluctuating between 69.95 ± 8.52 U/L to 71.92 ± 6.69 U/L. In the IN LNCs_Clo_ group, AST levels showed a slight increase during the treatment, with a final measurement of 88.33 ± 9.32 U/L in week 8. Therefore, the IN LNCs_Clo_ group displayed a more moderate and transient increase in AST levels, which stabilized over time and remained within a range closer to that of the control group (*p* > 0.05). In contrast, the oral Clo solution group, on the other hand, demonstrated a more pronounced and sustained increase in AST levels over time. By the third week, AST levels had risen to 101.36 ± 12.36 U/L and continued to climb to 132.66 ± 9.36 U/L by week 8, indicating a significant elevation in liver enzyme activity in response to oral Clo administration (*p* < 0.05). The results of this study reveal distinct effects of IN LNCs_Clo_ and the oral Clo solution on serum AST levels in rats. The substantial increase in AST levels observed in the oral Clo solution group is consistent with the known hepatotoxic effects of Clo, particularly when administered orally [60,61,62].

Elevated liver enzyme levels have been frequently reported in patients receiving oral Clo, and this study reinforces the need for careful monitoring of liver function during treatment. The ability of IN administration to bypass first-pass metabolism, thereby reducing systemic drug exposure, could account for the more favorable liver enzyme profile observed in this group. The impact of IN LNCs_Clo_ and the oral Clo solution on serum triglyceride on serum triglyceride levels in rats over an 8-week period is illustrated in Figure 4C. The control group showed minimal fluctuations in serum triglyceride levels throughout the study, with values remaining within a stable range of 4.69 ± 0.65 mmol/L to 7.11 ± 0.87 mmol/L, indicating no significant changes in lipid metabolism over the 8-week period. The IN LNCs_Clo_ group exhibited a moderate increase in triglyceride levels over time, peaking at week 5 with a mean value of 9.36 ± 1.03 mmol/L before gradually declining to 7.69 ± 0.69 mmol/L at week 8. The increase in triglyceride levels in the IN LNCs_Clo_ group, though present, was significantly less pronounced than in the oral clozapine group. The oral Clo solution group showed a substantial and consistent rise in triglyceride levels, beginning in the second week 2 with a mean value of 8.69 ± 1.02 mmol/L and reaching a peak of 18.36 ± 1.87 mmol/L by week 7. By week 8, the mean triglyceride level remained elevated at 15.36 ± 1.98 mmol/L, indicating a pronounced effect of oral Clo solution on lipid metabolism. Triglycerides are an essential marker of lipid metabolism, and elevated serum triglyceride levels are associated with an increased risk of cardiovascular disease and other metabolic disorders [63]. The marked rise in triglyceride levels observed in the oral Clo solution group is consistent with Clo’s known adverse effects on lipid metabolism. Oral Clo administration has been linked to hypertriglyceridemia in both clinical and preclinical settings, likely due to its influence on lipid regulatory pathways and its capacity to induce insulin resistance [64,65]. Therefore, the attenuated triglyceride response in this group supports the hypothesis that IN drug administration can limit systemic exposure and potentially mitigate some of the metabolic disturbances commonly associated with oral Clo administration.

### 3.4. Paw Placement Test

**Figure 5:**
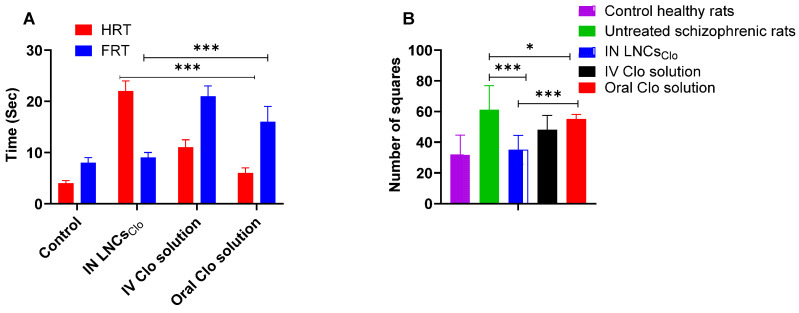
Assessment of the pharmacodynamics effects of intranasal LNCs_Clo_, IV Clo solution, and oral Clo solution using the (**A**) paw test and (**B**) ketamine-induced schizophrenia in rats via an open field test. A significantly higher anti-schizophrenic effect was observed following IN administration of LNCs_Clo_ compared to IV and oral Clo solutions. Datapoints represent mean ± SE (*n* = 10). One-way analysis of variance (ANOVA) was used to compare the different parameters between groups, followed by the Tukey HSD test, * *p* < 0.05, and *** *p* < 0.001.

Figure 5A shows the effects of IN LNCs_Clo_, IV Clo solution, and oral Cl solution on HRT and FRT compared to the untreated control animals using the paw test. The results of the paw test reveal distinct effects of IN, IV, and oral Clo formulations on motor response times. In the control group, the mean HRT was 4 ± 0.5 s, while the FRT was 8 ± 1 s. The IN LNCs_Clo_ group exhibited a significant increase in HRT, with a mean of 22 ± 2 s, indicating a notable delay in motor response (*p* < 0.001). However, the FRT in this group remained comparable to the control group, with a mean of 9 ± 1 s (*p* > 0.05), suggesting that the IN formulation had a more pronounced effect on hindlimb motor function than on forelimb function [38,66].

In the IV Clo group, both HRT and FRT were substantially elevated compared to the control group. The mean HRT was 11 ± 1.5 s, and the mean FRT was 21 ± 2 s. These findings indicate that IV administration of Clo significantly delayed motor responses in both the hindlimbs and forelimbs (*p* < 0.05). Moreover, the oral Clo solution group showed a moderate delay in motor response. The mean HRT was 6 ± 1 s, and the mean FRT was 16 ± 3 s, suggesting that oral Clo had a less pronounced effect on hindlimb function compared to IN administration but a greater impact on forelimb function compared to the control group. The significant prolongation of HRT observed in the IN LNCs_Clo_ group suggests that it may have a potent effect on motor coordination, particularly in the hindlimbs. The minimal impact on FRT in this group indicates that the motor impairment was more localized to hindlimb function. This could be attributed to the distribution of Clo following IN administration, which may preferentially affect certain neural circuits involved in hindlimb coordination [37,38]. In contrast, the IV Clo solution group displayed significant delays in both HRT and FRT, indicating widespread motor impairment. IV administration results in rapid systemic distribution, potentially leading to broader effects on motor control in both the hindlimbs and forelimbs. These findings underscore the varying impacts of different routes of Clo administration on motor function. IN administration appears to specifically impair hindlimb coordination, while IV and oral clozapine produce broader motor impairments affecting both the hindlimbs and forelimbs, which may reflect differences in the pharmacokinetics and distribution of Clo when administered orally versus intranasally.

### 3.5. Pharmacodynamic Assessment of LNCs_Clo_ in a Rat Model of Schizophrenia

To ensure the improvement of the antipsychotic effect of IN LNCs_Clo,_ schizophrenia-induced rats were used. The data presented in Figure 5B demonstrate that different Clo formulations have varying effects on locomotor activity in rats, with IN LNCs_Clo_ showing the highest efficacy in reducing hyperactivity associated with schizophrenia. Schizophrenia is often characterized by elevated locomotor activity, and the untreated schizophrenic rats in this study exhibited significantly higher activity levels than the control group. The untreated schizophrenic rats displayed a significantly increased locomotor activity, with a mean of 61 ± 5 squares, compared to the control group of healthy rats, which showed a baseline locomotor activity of 32 ± 4 squares. This increase in activity reflects the hyperactive behavioral phenotype commonly seen in animal models of schizophrenia [67]. The IN LNCs_Clo_ group showed a substantial reduction in hyperactivity (35 ± 3 squares), with activity levels approaching those of the control group (*p* > 0.05). This suggests that IN delivery of LNCs_Clo_ may offer an effective means of normalizing locomotor function without inducing excessive sedation. The high Clo EE% in the formulated LNCs with a particle size of 28.6 ± 3.6 nm is responsible for the improved therapeutic outcomes. Additionally, the surfactant present in the LNCs promotes the opening of endothelial tight junctions, transcytosis, and endocytosis, all of which boost Clo delivery to the brain [68]. The IV Clo solution group also demonstrated a reduction in locomotor activity to 48 ± 3 squares, although the effect was less pronounced compared to the IN LNCs_Clo_. This may be due to the more widespread distribution of Clo following IV administration, which could result in a less targeted reduction of hyperactivity. Furthermore, the oral Clo group exhibited the least reduction in hyperactivity reaching 55 ± 3 squares, suggesting that oral administration may be less effective at controlling schizophrenia-related hyperactivity. This could be related to the pharmacokinetics of oral Clo, which may lead to first-pass metabolism, resulting in less efficient control of hyperactivity [69]. These findings suggest that the use of IN LNCs_Clo_ to restore locomotor activity to near-normal levels highlights their potential therapeutic advantage.

## 4. Conclusions

This study successfully demonstrates the potential of IN administration of LNCs_Clo_ as an innovative approach for targeted brain delivery, offering an alternative to traditional oral and IV routes. The fabricated LNCs_Clo_ formulation had a favorable particle size, high EE%, and a sustained release profile, indicating its suitability for efficient brain targeting. Pharmacokinetic analysis revealed enhanced bioavailability, rapid brain delivery, and prolonged retention of Clo when administered intranasally, compared to oral and IV solutions. Additionally, the LNCs_Clo_ formulation exhibited a favorable safety profile, with less impact on plasma glucose, insulin, triglyceride, and liver enzymes, highlighting its potential to mitigate the metabolic and hepatotoxic side effects associated with Clo. The pharmacodynamic assessments further demonstrated significant improvement in antipsychotic efficacy and motor coordination, suggesting that IN LNCs_Clo_ could effectively normalize schizophrenia-associated behaviors. Overall, these findings underscore the promise of LNCs_Clo_ as a safer and more efficient drug delivery platform for the treatment of CNS disorders like schizophrenia, warranting further clinical investigation to validate its therapeutic potential.

## Figures and Tables

**Table 1:** Characterization of the prepared LNCs_Clo_ formulation.

LNCs_Clo_	Particle Size (nm) ^a,e^	PDI ^a,e^	Zeta Potential (mV) ^b,e^	EE % ^c,e^	LE% ^d,e^
Characterization	28.6 ± 3.6	0.18 ± 0.02	−15.9 ± 1.9	84.66 ± 5.66	17.65 ± 2.11

^a^ Measured by DLS. ^b^ Measured by electrophoresis. ^c^ Calculated as a percentage of entrapped Clo to the initial Clo added, determined directly by HPLC. ^d^ Calculated as a percentage of entrapped Clo weight to the total LNCs weight. ^e^ Expressed as mean ± SD (*n* = 3).

**Table 2:** Pharmacokinetic parameters of IN LNCs_Clo_ compared to IV and oral solutions.

Parameter	Plasma			Brain		
	LNCs_Clo_	IV Clo Solution	Oral Clo Solution	LNCs_Clo_	IV Clo Solution	Oral Clo Solution
C_max_ (ng/mL)	327.78 ± 33.66	--------	148.52 ± 22.54	498.09 ± 43.03	241.39 ± 32.34	60.62 ± 6.78
T_max_ (min)	60	--------	30	15	60	120
AUC_0–480min_ (ng/mL.h)	1101.8 ± 121.31	1677.23 ± 89.98	437.93 ± 46	2036.45 ± 120.03	951.78 ± 115.41	248.33 ± 42.21
MRT (h)	4 ± 0.25	4.23 ± 0.51	3.32 ± 0.11	4.75 ± 0.71	4.32 ± 0.58	2.64 ± 0.33
K_el_ (h^−1^)	0.18 ± 0.02	0.15 ± 0.01	0.25 ± 0.01	0.12 ± 0.01	0.16 ± 0.02	0.26 ± 0.02
Absolute bioavailability (F%)	65.69	--------	26.11	--------	--------	--------
DTE (%)	--------	--------	--------	325.71	--------	--------
DTP (%)	--------	--------	--------	69.29	--------	--------

## Data Availability

Data will be made available upon request.

## Supplementary Materials

The following supporting information can be downloaded at https://www.mdpi.com/article/10.3390/pharmaceutics16111417/s1, Figure S1: Calibration curve of Clo in PBS (pH 7.4) containing Triton X-100 (10%).; Figure S2: Calibration curve of Clo in rat plasma (A) and brain homogenate (B).
