# Supplementary material for: Manipulation of Lipid Nanocapsules as an Efficient Intranasal Platform for Brain Deposition of Clozapine as an Antipsychotic Drug
Source: Pharmaceutics. 2024 Nov 5;16(11):1417. doi: 10.3390/pharmaceutics16111417 (PMC11597305; doi:10.3390/pharmaceutics16111417)
Supplement: Supplementary file 1 [file pharmaceutics-16-01417-s001.zip › pharmaceutics-3256988-supplementary.pdf]

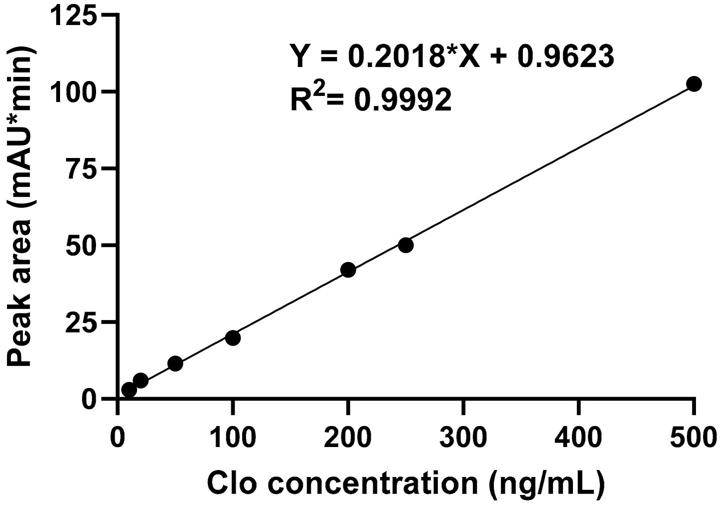

Figure S1. Calibration curve of Clo in PBS (pH 7.4) containing Triton X-100 (10%).

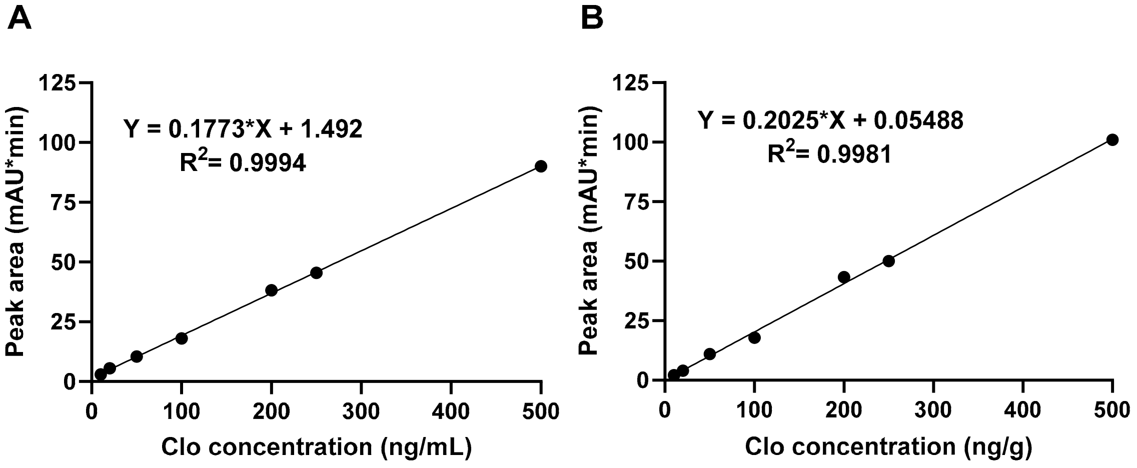

Figure S2. Calibration curve of Clo in rat plasma (A) and brain homogenate (B).
